# Quality of care: measuring a neglected driver of improved health

**DOI:** 10.2471/BLT.16.180190

**Published:** 2016-02-21

**Authors:** Yoko Akachi, Margaret E Kruk

**Affiliations:** a United Nations University World Institute for Development, Katajanokanlaituri 6B, FI-00160, Helsinki, Finland.; b Department of Global Health and Population, Harvard TH Chan School of Public Health, Boston, United States of America.

## Abstract

The quality of care provided by health systems contributes towards efforts to reach sustainable development goal 3 on health and well-being. There is growing evidence that the impact of health interventions is undermined by poor quality of care in lower-income countries. Quality of care will also be crucial to the success of universal health coverage initiatives; citizens unhappy with the quality and scope of covered services are unlikely to support public financing of health care. Moreover, an ethical impetus exists to ensure that all people, including the poorest, obtain a minimum quality standard of care that is effective for improving health. However, the measurement of quality today in low- and middle-income countries is inadequate to the task. Health information systems provide incomplete and often unreliable data, and facility surveys collect too many indicators of uncertain utility, focus on a limited number of services and are quickly out of date. Existing measures poorly capture the process of care and the patient experience. Patient outcomes that are sensitive to health-care practices, a mainstay of quality assessment in high-income countries, are rarely collected. We propose six policy recommendations to improve quality-of-care measurement and amplify its policy impact: (i) redouble efforts to improve and institutionalize civil registration and vital statistics systems; (ii) reform facility surveys and strengthen routine information systems; (iii) innovate new quality measures for low-resource contexts; (iv) get the patient perspective on quality; (v) invest in national quality data; and (vi) translate quality evidence for policy impact.

## Résumé

La qualité des soins dispensés par les systèmes de santé contribue aux efforts qui visent à atteindre l'objectif de développement durable n°3 sur la santé et le bien-être. De plus en plus d'éléments indiquent que l'impact des interventions dans le domaine de la santé est compromis par une mauvaise qualité des soins dans les pays aux revenus les plus faibles. La qualité des soins sera également cruciale pour la réussite des initiatives en faveur de la couverture sanitaire universelle; il est peu probable que des personnes insatisfaites de la qualité et de l'étendue des services proposés soient favorables au financement public des soins. De plus, l'éthique veut que toutes les personnes, même les plus pauvres, puissent bénéficier d'une prise en charge de qualité minimum qui leur permette de voir leur santé s'améliorer. Or, aujourd'hui, dans les pays à revenu faible et intermédiaire, l'évaluation de la qualité n'est pas à la hauteur de la tâche. Les systèmes d'information sanitaire fournissent des données incomplètes et souvent peu fiables. Quant aux enquêtes menées dans les établissements, elles recueillent trop d'indicateurs à l'utilité discutable, s'intéressent à un nombre limité de services et sont rapidement obsolètes. Les évaluations existantes n'intègrent pas bien le processus de soins et le vécu des patients. Le point de vue des patients sensibles aux pratiques de soins, un élément de base de l'évaluation de la qualité dans les pays à revenu élevé, est rarement recueilli. Nous proposons six recommandations stratégiques afin d'améliorer l'évaluation de la qualité des soins et d'amplifier son impact stratégique: (i) redoubler les efforts pour améliorer et institutionnaliser les systèmes d'enregistrement et de statistiques de l'état civil; (ii) réformer les enquêtes menées dans les établissements et renforcer les systèmes d'information courants; (iii) définir de nouvelles mesures de la qualité pour les régions aux faibles ressources; (iv) recueillir l'avis des patients concernant la qualité; (v) investir dans des données nationales de qualité; et (vi) traduire les données sur la qualité en actions ayant un impact stratégique.

## Resumen

La calidad de la atención proporcionada por los sistemas sanitarios contribuye a los esfuerzos para alcanzar el objetivo 3 de desarrollo sostenible en salud y bienestar. Cada vez existen más pruebas de que el impacto de las intervenciones sanitarias se ve desvirtuado por la mala calidad de la atención en países con ingresos bajos. La calidad de la salud también será fundamental para el éxito de las iniciativas de cobertura sanitaria universal; los ciudadanos que no están satisfechos con la calidad y el alcance de los servicios cubiertos no suelen apoyar la financiación pública de la atención sanitaria. Asimismo, existe un ímpetu ético para garantizar que todo el mundo, incluida la población más pobre, obtiene un estándar mínimo de calidad de atención que sea eficaz a la hora de mejorar la salud. No obstante, actualmente la medición de la calidad en países de ingresos bajos y medios no es adecuada para la tarea. Los sistemas de información sanitaria ofrecen información incompleta y a menudo poco fiable, y las encuestas en los centros recopilan demasiados indicadores de servicios públicos inestables, se centran en un número limitado de servicios y quedan obsoletos con mucha rapidez. Las medidas existentes apenas captan el proceso de atención y la experiencia del paciente. Los resultados de los pacientes sensibles a las prácticas de atención sanitaria son un elemento fundamental de la evaluación de la calidad en países con ingresos altos, y apenas se recopilan. Se proponen seis recomendaciones normativas para mejorar la medición de la calidad de la atención y ampliar el impacto de su política: (i) redoblar los esfuerzos para mejorar e institucionalizar los sistemas de registro civil y de estadísticas vitales; (ii) reformar las encuestas de los centros y fortalecer los sistemas de información rutinaria; (iii) crear nuevas medidas de calidad para contextos de pocos recursos; (iv) ofrecer al paciente una perspectiva sobre calidad; (v) invertir en datos de calidad nacionales; y (vi) traducir las pruebas de calidad para lograr un impacto de la política.

## ملخص

تساهم الجودة النوعية للرعاية المُقدمة من النظم الصحية في الجهود الرامية للوصول إلى الهدف الثالث (3) للتنمية المستدامة والمتعلق بالصحة والرفاهية. وهناك أدلة متزايدة تشير إلى ضعف دور التدخلات الصحية بسبب تردي الجودة النوعية للرعاية في البلدان ذات الدخل المنخفض. كما ستلعب الجودة النوعية للرعاية دورًا حاسمًا في نجاح مبادرات التغطية الصحية على المستوى العالمي، فليس من المتوقع أن يقوم مواطنون غير راضين عن الجودة النوعية ونطاق الخدمات التي يتم تغطيتها بدعم التمويل العمومي للرعاية الصحية. فضلًا عن ذلك فإن وجود الدافع الأخلاقي يعمل على ضمان حصول جميع الأشخاص – بمن فيهم الأشد فقرًا – على الحد الأدنى من الجودة النوعية للرعاية والتي تعتبر ضرورة لتحسين الحالة الصحية. على الرغم من ذلك فإن مقاييس الجودة النوعية القائمة اليوم في البلدان منخفضة ومتوسطة الدخل تعتبر غير ملائمة لهذا الدور، حيث توفر نظم المعلومات الصحية بيانات غير مكتملة وغالبًا غير موثوقة، وتعمل المسوحات التي تجري في المنشآت الطبية على جمع الكثير والكثير من المؤشرات مُبهمة الفائدة مع تركيز كل اهتمامها على عدد محدود من الخدمات والتي سريعًا ما ستصبح خارج إطار الزمن. كما تعجز المقاييس الحالية عن تناول عملية الرعاية والتجربة التي يعاشها المريض على النحو الأمثل. ونادرًا ما يتم جمع النتائج المتعلقة بالمرضى رغم أهميتها في مجال الممارسات المتبعة للرعاية الصحية ودورها كأساس لتقييم الجودة النوعية في البلدان مرتفعة الدخل. ونحن نقدم ست توصيات للسياسات العامة تهدف إلى تحسين مقياس الجودة النوعية للرعاية وتعظيم تأثيرها، وهي تتمثل في: (1) مضاعفة الجهود للعمل على تحسين نظم التسجيل المدني ونظم الإحصاءات الحيوية وتكريس استخدامها لدى المؤسسات، و(2) تطوير المسوحات المتبعة لدى المنشآت الطبية وتعزيز نظم المعلومات الروتينية، و(3) ابتكار مقاييس جودة جديدة للبيئات شحيحة الموارد، و(4) استطلاع آراء المرضى حول الجودة النوعية للرعاية، و(5) الاستثمار في جمع بيانات ذات جودة نوعية رفيعة على المستوى الوطني، و(6) تفسير مؤشرات الجودة النوعية لتعظيم أثر السياسات العامة.

## 摘要

由卫生系统提供的护理质量影响健康与幸福方面的可持续发展目标 3 的实现。 越来越多证据表明，卫生干预措施的影响受到了低收入国家较差护理质量的破坏。 护理质量对于全民卫生覆盖活动的成功同样至关重要；对普及服务的质量及范围不满的市民不可能支持公共医疗保健融资。 此外，道德推力的存在将确保所有人（包括最贫困人员）均可获得可有效改善健康的最低质量标准的护理。 然而，中低收入国家现今的质量衡量结果仍不足以完成该任务。 卫生信息系统提供不完整且通常不可靠的数据，机构调查采集过多用途不确定的指标，专注在有限的服务上并且很快就会过期。 现有量具无法准确收集护理流程及患者体验的数据。 几乎很少收集对医疗保健实践敏感的患者效果，而这一指标是高收入国家质量评估的重要度量。 我们提出了六项政策建议以改善护理衡量的质量并扩大该政策的影响： (i) 加倍努力改善民事登记及生命数据系统，并使其制度化；(ii) 改革机构调查并加强常规信息系统；(iii) 向资源匮乏地区引进新的质量量具；(iv) 获取患者对质量的看法；(v) 投资全国质量数据；以及 (vi) 转化质量证据以获取政策影响。

## Резюме

Качество медицинской помощи, обеспечиваемое системами здравоохранения, вносит свой вклад в усилия по достижению цели 3 в области устойчивого развития, направленной на улучшение здоровья и благополучия. Все больше фактов свидетельствуют о том, что эффект проведения мероприятий в области здравоохранения нивелируется плохим качеством медицинской помощи в странах с низким уровнем дохода. Качество медицинской помощи также будет иметь решающее значение для успеха инициатив по всеобщему охвату услугами систем здравоохранения; граждане, недовольные качеством и содержанием предоставляемых услуг, вряд ли будут поддерживать государственное финансирование здравоохранения. Кроме того, существует этический стимул для гарантии того, что все люди, в том числе самые бедные, смогут получить минимальный стандарт качества медицинского обслуживания, который является эффективным для улучшения состояния здоровья. Тем не менее оценка качества сегодня в странах с низким и средним уровнем дохода является недостаточной для выполнения этой задачи. Информационные системы здравоохранения предоставляют неполные и часто недостоверные данные, а в обследованиях учреждений применяется слишком много показателей неопределенной полезности и уделяется внимание ограниченному ряду услуг. Кроме того, такие обследования быстро устаревают. Существующие оценки отражают процесс медицинского ухода и впечатления пациента не в полной мере. Результаты ведения пациентов, зависящие от принятых методов в сфере здравоохранения и являющиеся основным показателем качества в странах с высоким уровнем дохода, редко собираются. Авторы предлагают шесть рекомендаций в отношении политики, призванных усовершенствовать оценку качества медицинской помощи и усилить эффект этой политики: (i) удвоить усилия по усовершенствованию и официальному закреплению систем регистрации актов гражданского состояния и статистики естественного движения населения; (ii) реорганизовать обследования учреждений и улучшить стандартные информационные системы; (iii) внедрить новые оценки качества для условий ограниченности ресурсов; (iv) взглянуть на качество с точки зрения пациента; (v) инвестировать в данные о качестве на национальном уровне; (vi) использовать фактические данные о качестве для усиления эффекта политики.

## Introduction

High quality of health care is an important component of efforts to reach sustainable development goal (SDG) 3: to ensure healthy lives and promote well-being for all at all ages.^1^The United States National Academy of Medicine defines quality as the extent to which health-care services provided to individuals and patient populations improve desired health outcomes.^2^ The key tasks for quality measurement are to assess the performance of services and to quantify the gap between reality and expectations in reference to certain standards and guidelines. However, a lack of consensus exists on the role of quality of care in achieving SDG 3,^3^ which is reflected in the absence of measures of quality that are appropriate to lower-income settings. This paper addresses the rationale for elevating the issue of quality in the global health discourse. We outline the current status of quality measurement in low- and middle-income countries and the gaps in measuring quality of care. We conclude with policy recommendations.

## Why now?

For the following reasons we propose that now is the time to focus on quality of care in low-resource settings and, specifically, to tackle the challenges of measurement. 

### Responding to complexity

The millennium development goals (MDGs) on health focused on combating maternal and child mortality and a relatively small number of diseases.^4^ These efforts boosted disease-specific (vertical) funding for health services and in some cases were accompanied by strong accountability mechanisms including measurement of outcomes and service quality.^5^ SDG 3 and its targets encompass more conditions, and, by including noncommunicable diseases, are also more complex to attain than the MDGs. As we move into the SDG era, the funding and delivery streams are being interconnected and integrated into broader health systems to promote more rational and patient-centred health care across a wide range of health needs. This is observed at both global^6^and country levels. The logistics of integration, including ensuring technical efficiency, will be challenging, but may also provide an opportunity for adoption of best practices in quality management in areas ranging from stand-alone vertical programmes to the broader health system.^7^

### Acting on evidence

The impact of health-service quality on health outcomes has been well documented in high-income countries,^8^^–^^11^ and poor quality is increasingly being linked to failure to attain expected health-care improvements in low- and middle-income countries. Studies from India, Malawi and Rwanda have shown that greater access to institutional deliveries and antenatal care was not accompanied by reductions in maternal and newborn mortality; a finding attributed to poor quality of care.^12^^–^^15^ Higher than predicted maternal mortality has been found in hospitals in high-mortality lower-income countries, despite good availability of essential medicines, suggesting clinical management gaps or treatment delays for women who develop obstetric complications.^12^ In the area of infectious disease control, nearly one third of all outpatients (*n* = 2019) in publicly-funded health facilities in Malawi received incorrect malaria treatment.^16^ Providers in India frequently gave inaccurate care to tuberculosis patients;^17^ in one study only 11 of 201 private practitioners followed diagnostic standards for tuberculosis management.^18^ Worldwide, low-quality facilities have been implicated in higher mortality after surgery.^19^ The effects of low quality of health services will be magnified in efforts to achieve the more complex SDG health goals.^3^

### Ensuring policy success

Quality of care is also central to the success of several health policy instruments recently introduced in low- and middle-income countries, such as universal health coverage and results-based financing. The universal health coverage target of SDG 3 (target 3.8) requires that everyone have access to affordable and quality health services. But if those services are poor quality, people are unlikely to use them or agree to pay higher taxes or insurance premiums for them. Most countries in Latin America, for example, have explicit provisions in their constitutions guaranteeing the right to health care for all citizens, and many nations have embarked on universal health coverage.^20^ However, in Mexico, the effective or quality-corrected coverage of health services is relatively low, and varies widely across states, despite achievement of universal health coverage.^21^ Furthermore, Mexicans have high out-of-pocket expenditures, partly due to using private health care to supplement the public system.^22^ If universal health coverage fails to provide high-quality services, those who can afford it will choose to seek care outside the system, thus undermining public support for – and the sustainability of – financing of universal health coverage. The quality of health-care services funded by universal health coverage needs to be monitored and if necessary, improved, to promote appropriate utilization, stable financing and better outcomes. Results-based financing, called pay-for-performance in high-income settings, is increasingly being used to expand the use and quality of specific health services in low- and middle-income countries. While results-based financing has increased the use of some health services through performance-based incentives to health workers, evidence of its impact on quality is inconclusive.^23^^,^^24^ More and better research is required to know whether this can be a useful instrument for improving quality and attaining desired health outcomes.

### Resolving ethical concerns

There is also an ethical dimension to quality of care. While the right to health care is widely accepted, less has been said about the quality of this care. First, one of the core principles of medicine is to do no harm. Yet there is still minimal systematic measurement of patient safety in the health systems of low- and middle-income countries.^25^^–^^27^ Second, little is known about wealth inequalities in the quality of care received. Julian Tudor Hart famously noted that the availability of good medical care tends to vary inversely with the need of the population served.^28^ We do not know how the quality of services available to poor people compares with that of richer people in the same country. The quality of care should be monitored and evaluated regardless of who provides the care, i.e. equally in private and public settings, and for both curative and preventive care. The work on equity of coverage led by the Countdown to 2015 initiative provides an excellent model for analysis and policy translation of equity data that can be adapted to quality of care.^29^

A third ethical issue is defining the quality baseline. What are appropriate quality standards in countries with extremely constrained health resources? Should doctors in remote African villages follow the same guidelines in treating fever as those used in North American medical centres? Some argue that less effective care is ethically acceptable when the alternative is no care, but this assumes that the care will still bring substantial benefit to patients.^30^ What is the minimum effectiveness that we are willing to accept, given the costs of health-care provision to governments and to families, and the legitimate expectations of people receiving the care? Finally, once a minimum standard is defined, the pursuit of a higher level of quality must be balanced with its cost and with the need to guarantee the minimum level of care quality to the entire population.^31^ Countries will need to define a quality frontier that situates their aspirations for quality within realistic budget constraints and that recognizes trade-offs between speed of expanding services and ensuring minimum quality standards. For this, countries require detailed data on the cost of quality improvement strategies: data that do not exist today.

## Current status

**Fig. 1:**
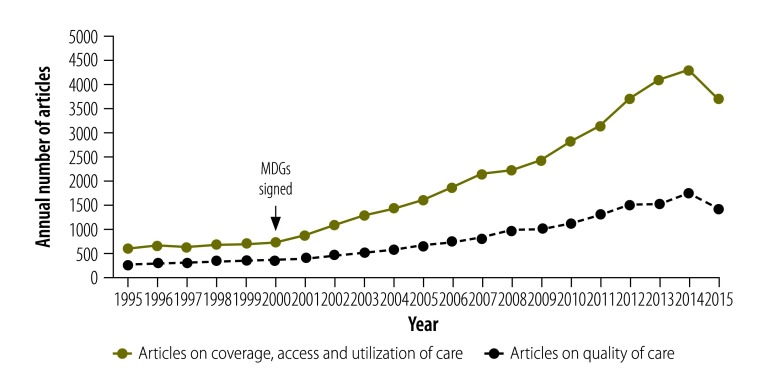
Annual number of articles published on quality and coverage of health care from the countries of Asia, Africa and Latin America, 1995–2015

What was the status of quality-of-care measurement in 2016? Systematic assessments of quality of care conducted in Europe and the United States of America in the early 2000s ushered in a new era of quality measurement and quality improvement in high-income countries.^8^^,^^10^^,^^11^ This was not matched, however, in lower-income countries. Although the signing of the MDGs in the year 2000 led to an explosion of measurement and research on coverage, access and utilization of health care in low- and middle-income countries, a similar pattern was not observed for research on quality of care. This is illustrated by a simple search of the PubMed database for the number of articles published on these topics from African, Asian or Latin American countries over the years 1995‒2015 (Fig. 1).

MDG: millennium development goals.

Notes: An online search of the PubMed database was made (by the authors) on 17 May 2016 for articles matching the terms “Asia” or “Africa” or “Latin America”. Using the results by year option in PubMed, we downloaded data (comma-separated value files) on the number of articles listed per year and converted these to a chart. The solid line shows articles indexed under “coverage” or “utilization” or “access.” The dotted line shows articles indexed under “quality of care”.

**Fig. 2:**
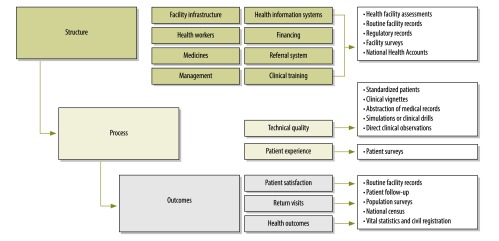
Domains of quality-of-care measurement and data sources

While individual projects in lower-income countries frequently collect data on quality of care, there has been relatively little research that can permit comparison and benchmarking of quality within or across countries. A framework for the different ways to measure quality is presented in Fig. 2. Following Donabedian’s theory of quality of care,^31^ the framework proposes three dimensions of quality of care that need to be tracked and, ideally, linked: (i) structure (facility infrastructure, management and staffing), (ii) process (technical [clinical] quality and patient experience) and (iii) outcomes (patient satisfaction, return visits and health outcomes). In high-income countries the main measures of quality have typically been patient outcomes that are sensitive to health-care practices, such as the association between skilled nursing and hospital readmissions.^32^^–^^34^ Nevertheless, there are calls to reconsider the importance of process measures that can provide concrete guidance on where to begin improvement efforts.^35^ Since many low- and middle-income countries lack the health information systems to collect these care-sensitive outcome measures, it is reasonable to begin with inputs and process measures. Inputs, such as water, sanitation and electricity, represent the minimum threshold for a functioning health-care facility; this is sometimes termed service readiness. Most of the existing efforts to measure quality have emphasized this tangible element of care, yet a cabinet full of unexpired medicines does not necessarily translate into good clinical care, and the connection between inputs and processes is poorly understood. Much more emphasis is needed on measuring the processes of care ‒ the content and nature of clinical interactions ‒ and the intangible elements of care underlying those interactions ‒ such as health-sector organization, facility management and staff training and motivation. This is especially timely as it relates to ongoing debates on task-shifting of health care from physicians to non-physician health workers.^36^ Ultimately, we need evidence linking quality of care to health outcomes, and this is why the benchmarking of quality of care in the specific context of low- and middle-income countries is necessary.

## Policy gaps

Given the constrained resources, it is essential for the quality-of-care measurement framework to prioritize the questions asked to identify the limitations on what is being done.

### Structure

Data for measuring the structure dimension of quality care, including facility infrastructure, staffing and clinical training, generally come from routine health-facility records and surveys. Record systems suffer from incomplete and inaccurate data as well as reporting delays, often resulting in out-of-date information of little use. Routinely collected health data are not standardized, precluding comparison across and, sometimes, within countries.^37^^–^^39^ Periodic health-facility surveys can provide better quality data, but such surveys describe the situation at one point in time and are restricted to a few services, typically excluding noncommunicable diseases, injuries and mental health, for example. A recent comprehensive review of health-facility assessment tools in low- and middle-income countries found that among the 10 tools that met the study´s inclusion criteria there was substantial variation in their content and comprehensiveness. Of the 41 domains for comprehensive health system measurement identified by the authors, the actual number of domains covered by each of the assessment tools ranged from 13 to 33, with a median of 25.5.^40^ For example, even when data on the health workforce were available, the indicators on staff presence and availability of emergency staff were mostly missing, as were any assessments of the clinical training the staff had received.^40^ The review raised two concerns; first, the data being collected at the health facility level are inconsistent, incomplete and difficult to compare; and second, there is a preference towards the evaluation of primary-care services over those of secondary and tertiary care.^40^ Finally, facility surveys offer an incomplete picture of the state of health services. For example, one of the most widely implemented programme of surveys ‒ service provision assessments ‒ has been conducted in only a handful of countries and typically only once in each country. One positive step in this direction is the Health Data Collaborative’s efforts to revise and harmonize existing surveys to reduce duplication.^41^

### Process

Measures of process quality of health care include both its technical quality and the experience of the patients receiving the care. The tools available for assessment of provision of clinical care include standardized patients, clinical vignettes, abstraction of medical records, simulations or clinical drills, and direct clinical observations.^42^ Standardized patients are trained actors who make an unannounced visit to a health-care facility and present symptoms of a simulated condition; they complete an assessment checklist on the clinical actions of the provider after the visit.^42^ In clinical vignettes, practitioners follow a written clinical case, responding to questions that replicate certain stages of an actual clinic visit, such as taking a history, ordering tests and prescribing a treatment plan. Providers’ responses are scored against evidence-based criteria for managing the simulated disease.^43^ Abstraction of medical records to identify standards-based practice is a common way of evaluating clinical performance; however, its validity is undermined by the lack and inconsistency of records in resource-constrained settings. Also these data are often collected by trained health personnel, making it an expensive tasks.^42^ Audits, such as morbidity and mortality reviews, can also provide valuable insights into quality failures. Simulation and clinical drills, in which the practitioners are given a scenario and are instructed to demonstrate clinical skills on a mannequin, are mainly used for teaching rather than for assessing quality in practice. Clinical observation is the direct observation or recording of a real-life patient and is an effective, well-established method for evaluation. Clinical observation and standardized patients are considered to be the gold standard measures but they are resource-intensive methods and thus difficult to scale up. They also have limited utility for assessing the care of serious conditions that are either too rare to reliably observe or cannot be simulated by an actor.^44^

Another issue is that interpersonal care quality and the patient experience are rarely measured. Yet respectful treatment, convenience and good communication are important to patients as individuals and are needed for promoting greater adherence to treatment and better health outcomes.^45^ Respectful care, for example, plays an important role in improving patient satisfaction and encouraging return visits,^46^ and there is a need for this concept to be incorporated into broader quality measurement and improvement efforts. The scope of inquiry into drivers of quality must extend beyond the facility and the immediate health-care team; good quality depends on district-wide service organization, pre-service training and community accountability mechanisms, among many other factors. To understand the root causes of quality gaps, whether for technical or non-technical quality, it is necessary to obtain perspectives on quality from a range of health-system stakeholders. Face-to-face interviews with patients, and written surveys, are typically used to measure the patient experience. Patients are best-positioned to determine whether care aligns with their values and preferences, and to convey their experience of provider communication, service convenience and so on.^47^ The expansion of communication technology and social media provides new opportunities for getting feedback on quality of care and returning relevant information back to users.

### Outcomes

Care-sensitive outcomes have been the mainstay of quality measurement in wealthy countries. However, obtaining these data is costly as it requires follow-up of patients after facility visits, and is challenging in low-income settings which may lack systematic collection of population data. A first step would be to improve the collection of in-facility health outcomes, such as rates of surgical and maternal deaths, stillbirths and early newborn deaths. For this, routine health information systems need to be upgraded: an investment which has many health system benefits.^48^ Population-based health information sources, such as household surveys, censuses and civil registration or vital statistics, need to be strengthened to obtain data on health outcomes that can be linked to the quality of care provided in the health system. In particular, vital statistics are essential to understand the size and location of populations that require health services. Age- and sex-disaggregated population data permit calculation of effective coverage, which is a quality-corrected measure of population coverage of interventions and services and which can uncover gaps in care quality in the country.^49^ For example, an evaluation of the quality of routine and emergency intrapartum and postnatal care in Ghana found that although 68% of 15 884 women delivered in a health facility, the estimated effective coverage with high-quality obstetric care was only 18%.^50^

## Recommendations

As the above discussion notes, the status of quality-of-care measurement today is not adequate to guide countries committed to pursuing SDG 3 on health and well-being. We propose six recommendations to improve the measurement of quality of care and its impact on improving health outcomes in lower-income countries. These fall into three areas: improving data collection methods and instruments; expanding the scope of measurements; and translating the data for policy impact. The six recommendations are: (i) redouble efforts to improve and institutionalize civil registration and vital statistics systems; (ii) reform facility surveys and strengthen routine information systems; (iii) innovate new quality measures for low-resource contexts; (iv) get the patient perspective on quality; (v) invest in national quality-of-care data; and (vi) translate quality evidence for policy impact, and are presented in detail in Box 1.

Box 1 **Policy recommendations to improve quality-of-care measurement**

**Recommendations for improving data collection methods and instruments**

1. Redouble efforts to improve and institutionalize civil registration and vital statistics systems. Without an accurate count of all births and deaths, there is no accountability for health-system performance and no denominator for tracking health-care quality.

2. Reform facility surveys and strengthen routine health information systems. Current health-facility surveys need to be more concise, more frequent and more focused on processes and outcomes of care instead of inputs. Routine health information systems should be strengthened to collect accurate in-facility health outcomes. Strong routine information systems can be used to track quality over time and to evaluate improvement efforts.

3. Innovate new quality-of-care measures for low-resource contexts. Current outcome measures for conditions sensitive to health-care practices, and observation of clinical care, are not feasible for routine quality assessment in the lowest income countries. Development and validation of new measures and new measurement technologies are needed.

**Recommendation for expanding the scope of measurements**

4. Get the patient perspective on quality. Quality is too often seen as a supply-side concern. Yet patients form their own, highly relevant assessments of quality that affect their use of care and adherence to treatment and, ultimately, population health outcomes. Understanding the patient experience gives direct insight into what is and is not working towards achieving high quality of care.

**Recommendations for translating the data for policy impact**

5. Invest in national quality-of-care data. Rigorous collection of quality-of-care data must move beyond individual projects and facilities to the entire health system. Measurements that are representative at the national and subnational levels permit governments to plan and track improvement. The experience of the Countdown to 2015 initiative^29^ and similar efforts during the era of the MDGs showed the power of systematic, accurate national data in spurring action to improve health-care coverage. The same can be done for quality. Cross-national comparisons can create peer pressure among countries to improve health-system performance.

6. Translate quality evidence for policy impact. Robust and meaningful data presented in intuitive ways will greatly improve policy uptake of quality data. Global health funders should invest in national capacity to analyse and present data on cross-national collaborations on quality analysis as key public goods.

While countries themselves will need to take the lead, global partners can lend their experience, funds and technical support to develop new methods and disseminate robust, comparable statistics on quality of health care.
